# Design of PEI and Amine Modified Metakaolin-Brushite Hybrid Polymeric Composite Materials for CO_2_ Capturing

**DOI:** 10.3390/polym15071669

**Published:** 2023-03-27

**Authors:** Miljana Mirković, Muge Sari Yilmaz, Ljiljana Kljajević, Vladimir Pavlović, Marija Ivanović, Dunja Djukić, Tarik Eren

**Affiliations:** 1 Department of Materials, VINČA, Institute of Nuclear Sciences—National Institute of the Republic of Serbia, University of Belgrade, 11000 Belgrade, Serbia; 2 Chemical Engineering Department, Yildiz Technical University, 34220 Istanbul, Turkey; 3 Faculty of Agriculture, University of Belgrade, 11080 Belgrade, Serbia; 4 Institute of Technical Sciences of the Serbian Academy of Sciences and Arts, University of Belgrade, 11000 Belgrade, Serbia; 5 Faculty of Biology, University of Belgrade, 11000 Belgrade, Serbia; 6 Chemistry Department, Yildiz Technical University, 34220 Istanbul, Turkey

**Keywords:** hybrid geopolymers, PEI, XRD, SEM, CO_2_ capturing

## Abstract

In this paper, the properties of organic-inorganic hybrid polymer materials, which were synthesized from an aluminosilicate inorganic matrix with the addition of brushite and aminosilane grafted on one side and PEI covalently bonded composites on the other side, were examined. The synthesized organic-inorganic hybrid polymers were examined in terms of a structural, morphological, thermo-gravimetric, and adsorption-desorption analysis and also as potential CO_2_ capturers. The structural and phase properties as well as the percentage contents of the crystalline and amorphous phase were determined by the X-ray diffraction method. The higher content of the amorphous phase in the structure of hybrid polymers was proven in metakaolin and metakaolin-brushite hybrid samples with the addition of amino silane and with 1,000,000 PEI in a structure. The DRIFT method showed the main band changes with the addition of an organic phase and inorganic matrix. Microstructural studies with the EDS analysis showed a uniform distribution of organic and inorganic phases in the hybrid geopolymers. The thermo-gravimetric analysis showed that organic compounds are successfully bonded to inorganic polymer matrix, while adsorption-desorption analysis confirmed that the organic phase completely covered the surface of the inorganic matrix. The CO_2_ adsorption experiments showed that the amine-modified composites have the higher capture capacity, which is 0.685 mmol·g^−1^ for the GM10 sample and 0.581 mmol·g^−1^ for the BGM10 sample, with 1,000,000 PEI in the structure.

## 1. Introduction

It is clear that the release and accumulation of large amounts of carbon dioxide affects the creation of the greenhouse and, as one of the main pollutants, affects the quality of life of a large number of people worldwide. Using non-renewable energy sources, such as coal but also fuel, has led to carbon dioxide emissions increasing significantly over the last decade [1,2]. The need to find new materials and remove pollutants from the atmosphere has been increasingly in demand in the last couple of decades. Regarding this problem in the last few decades, organic-inorganic hybrid materials have been attracting more attention from researchers due to the interconnection of both properties, which is why they can be used in areas that require high-performance materials with a multifunctional design [3,4,5]. The key role of the organic-inorganic hybrids is in developing advanced functional materials because their chemical and physical properties are unique and stem from the synergistic interaction of organic and inorganic phases [6]. The inorganic component of the hybrid material (inorganic polymer) would be of aluminosilicate and calcium phosphate origin and would be based on local potential raw materials, such as kaolin clay, with materials from the calcium phosphate group [7]. Calcium phosphate materials and clay minerals are typical and fragile cement-based materials that can be modified by incorporating organic polymer to enhance the workability, flexural and tensile strength, and water resistance [8]. Therefore, lately, the synthesis of new hybrid organic-inorganic polymer materials has been increasing, where the main principle of synthesis is activating clay materials, creating the Si-Al-O polymer network, and adding an organic polymer coating, which creates a potential application for these materials in some new fields [9,10,11]. On the other hand, calcium phosphate materials, such as brushite and hydroxyapatite, are also bio-based materials whose excellent characteristics allow their usage in various fields such as biomedicines, drug delivery, and environmental remediation to be used as fillers in an inorganic matrix-thrown hybrid polymer preparation [12]. Additionally, due to their excellent structural characteristics, these materials can be used as adsorbents in various applications for organic and inorganic pollutants [13]. There is the possibility to use different type’s aluminosilicate materials as raw materials with phosphate types of cement for producing new inorganic polymer materials for specific applications [7]. Inorganic polymers based on natural aluminosilicate precursors with calcium phosphate are a relatively new class of materials [14,15], adding organic components to improve their materials’ mechanical properties, durability, and thermal stability. As an organic component of a hybrid material, it is most common in the literature: epoxy resins, polyethylene glycol-PEG, polyvinyl alcohol-PVA, silicone resins, etc. By synthesis of organic/inorganic hybrid materials, it is possible to achieve interfacial mixing where the inorganic hybrid material component contributes to a higher hardness and thermal stability, while the organic one may increase the elasticity and toughness of the finally obtained hybrid material [16,17,18].

Aluminosilicate matrices used from raw materials with the addition of phosphate cement as fillers, on the one hand, and covalently bonded organic compounds, on the other hand, are some of the innovations designed for obtaining organic-inorganic hybrid polymer materials. Designing the process from preparation to the application of such hybrid materials is a challenge due to requests to obtain materials with the potential usage of these materials as carbon dioxide catchers. In the existing literature, it is rare to find a geopolymer material that has been synthesized from a raw material with the addition of calcium phosphate cement, which was used as an inorganic matrix for attaching organic polymers. Since knowing the properties of inorganic polymer materials, the idea of this work was the design of new hybrid organic-inorganic materials that can be used for CO_2_ capture.

The main idea of our research is a synthesis of hybrid organic-inorganic polymer materials, which is simple and consists of the direct synthesis of an inorganic polymer paste, which further hardens itself in an organic solution, thus creating an organic-inorganic hybrid composite. In addition, due to less energy loss, which is required for their production, and high sustainability processes, there is great interest in their synthesis. The aim of this work was to include the application of newly designed hybrid organic-inorganic polymers as CO_2_ catchers.

## 2. Materials and Methods

Inorganic polymers (filler) are synthesized by activating inorganic precursors mainly consisting of silicon dioxide, aluminum, and low calcium oxide content, with the addition of phosphate types of cement in different weight percentages (%_w.t._). The previously investigated raw metakaolin clay (Kaolinite originates from Rudovci, Serbia) [19] was used as Al and Si sources for geopolymer synthesis. Brushite powder was synthesized by solution precipitation method by dropwise addition of 0.2 M calcium-acetate solution ((CH_3_COO)_2_Ca·H_2_O, Sigma Aldrich, p.a., St. Louis, MO, USA) to a 0.2 M sodium dihydrogen phosphate solution (NaH_2_PO_4_·H_2_O, Sigma Aldrich, p.a.) in equal volume. Solutions were stirred magnetically, at 60 °C, for one hour. After synthesis precipitate was washed in distilled water and dried overnight at 40°C. Two types of inorganic polymers are synthesized: GM—metakaolin geopolymer; BGM—metakaolin geopolymer with 2%_w.t._ of brushite in the structure. GM samples are synthesized by activation of metakaolin clay with 8 M alkaline solutions (NaOH, *Sigma Aldrich, p.a.*) and sodium silicate solution (analytical grade) in relation 1:1.6. BGM samples are synthesized in the same way with the addition of 2%_w.t_. of brushite in the metakaolin during synthesis. The process of consolidation of the activated precursor takes place at room temperature at temperatures of about 60 °C. The samples are aged at room temperature for 28 days.

To investigate the CO_2_ capture capacities, GM and BGM samples were modified with three different amine solutions. These are listed below.

**Figure 1:**
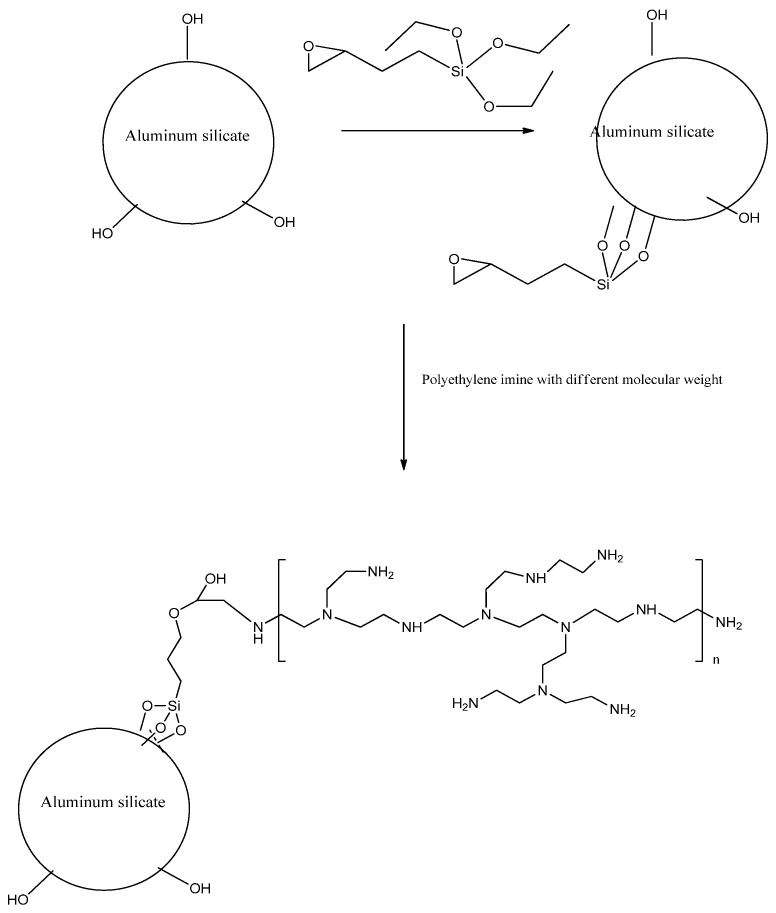
Scheme of surface modification of GM and BGM with organic compounds.

(i) *Preparation of different molecular weight of PEI covalently bonded composites*: GM and BGM composites are first modified with epoxy silane before being covalently bonded with PEI. In a typical procedure, glycidoxypropyl-functionalized composites were prepared by refluxing 2 g of the composite with 10 mL of 3-glycidoxypropyltrimethoxy-silane in 100 mL of dry toluene for 24 h in nitrogen. The resulting colloidal surface-coated composites were isolated and purified by centrifugation/redispersion in ethanol processes (for 10 min at 15,000 rpm, 5 times) to remove excess of 3-glycidoxypropyltrimethoxy-silane. The resulting solid was dried at room temperature under a vacuum for 24 h. Then, the obtained epoxy-grafted composites were modified with two different types of PEI samples (Molecular weight: 1,000,000 and 600). Therefore, 2 g PEI was first dissolved in 25 mL chloroform after stirring for 15 min, then 1 g of epoxy-grafted composite was added into the solution. The mixture was continuously stirred at 60 °C for 48 h and then purified by centrifugation/redispersion in chloroform processes (for 2 min at 6000 rpm, 5 times) to remove any excess PEI. The GM composites modified with 1,000,000 and 600 were denoted as GM10 and GM6. The BGM composites modified with 1,000,000 and 600 were denoted as BGM10 and BGM6. An illustration of the surface modification of GM and BGM with PEI is given in Figure 1.
(ii) *Preparation of aminosilane-grafted composites*: N1-(3-Trimethoxysilylpropyl) diethylenetriamine (TMPTA) was used to graft to the surfaces of the composites. A defined amount of composite was dissolved in dried toluene under ultrasonic irradiation for 3 h. The solution was kept at room temperature overnight. The obtained suspension was allowed to react with TMPTA under a nitrogen atmosphere at 50 °C for 5 h. The resulting solid product was separated by filtration, washed with toluene, and dried in a vacuum. The obtained samples were denoted as GM2 and BGM2.

The prepared sample composition is presented in Table 1.

The CO_2_ capture performances of polymer composites at different temperatures were determined using a Perkin Elmer Pyris Diamond thermogravimetric (TG) analyzer. First, approximately 9 mg of the sample was kept at 105 °C under a nitrogen atmosphere to remove moisture from the sample structure. Then, the temperature was adjusted to the desired temperature with a cooling rate of 10 °C/min and the gas flow was switched to CO_2_ (purity > 99.99%) and kept for a certain period. CO_2_ capture capacities of the samples were calculated via weight increase in the sample.

The polymer composite samples’ phase and crystallinity were performed by X-ray diffraction (XRD) analysis on an Ultima IV Rigaku diffractometer (Rigaku, Tokyo, Japan) equipped with copper (Cu) X-ray tube anode, CuK*α*_1,2_ radiation. The samples were powdered to ultra-fine grains (below 2 μm) in a ceramic mortar and placed on a monocrystalline silicon carrier. The recording was performed under a voltage of 40.0 kV and a generator current of 40.0 mA. The applied recording range was from 5 to 60° 2*θ*, with a step of 0.02°, and a recording speed of 5°/min using a D/TeX Ultra-fast detector. All the measurements were performed at room temperature. For phase analysis and identification, PDXL2 software was used [20] and equipped with ICDD crystallographic database; PDF card number 01-079-1910 was used for quartz identification; and PDF card number 00-046-0740 was used for paragonite identification [21].

The specific surface area (S_BET_) of samples GM2, GM 6, GM 10, BGM 2, BGM 6, and BGM 10 were analyzed using the Surfer (Thermo Fisher Scientific, Waltham, MA, USA). Before analyses, the samples were degassed at 105 °C for 4 h under a vacuum.

The physicochemical characterization of the synthesized geopolymer materials was performed using Diffuse Reflectance Infrared Fourier Transform (DRIFT) method (Perkin Elmer Spectrum Quant instrument, Beaconsfield, UK). Samples were dusted and evenly dispersed in anhydrous potassium bromide (KBr) pellets. Spectra were obtained at room temperature using and spectral data of the samples were collected in the region from 500 to 4000 cm^−1^.

Morphological properties of materials and semi-quantitative elemental analysis were performed using SEM-EDS analysis. All samples were Au-coated and examined using the JEOL JSM 6390 LV electron microscope at 30 kV.

Comparative thermal analysis and CO_2_ capturing analysis of bare, epoxy-silane-modified and PEI covalently bonded composites were performed on the same TG instrument (Perkin Elmer, Waltham, MA, USA) to prove the bonding of PEI to GM and BGM composites. The operating conditions were at a temperature of 25–600 °C in a nitrogen atmosphere, and at 10 °C.min^−1^ heating rate. The CO_2_ capture analysis of all the samples was carried out at 75 °C.

## 3. Results and Discussion

### 3.1. XRD Analysis

**Figure 2:**
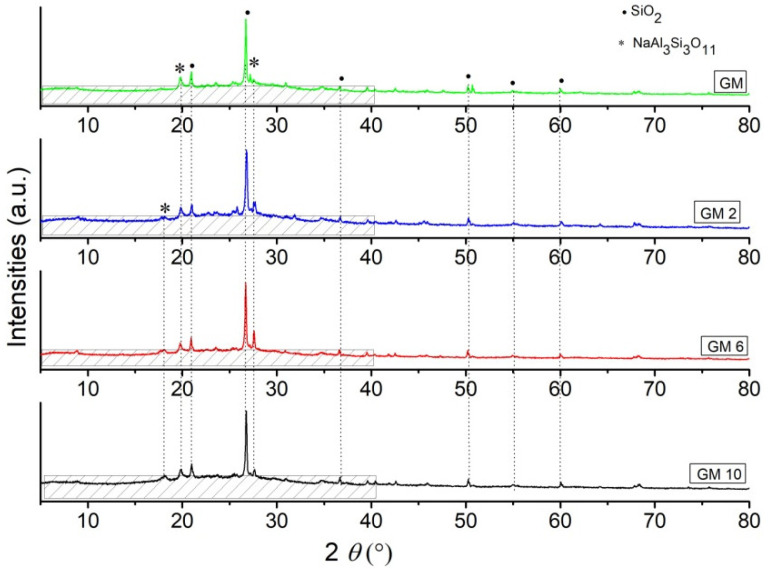
XRD diffraction patterns of GM, GM2, GM6, and GM10.

**Figure 3:**
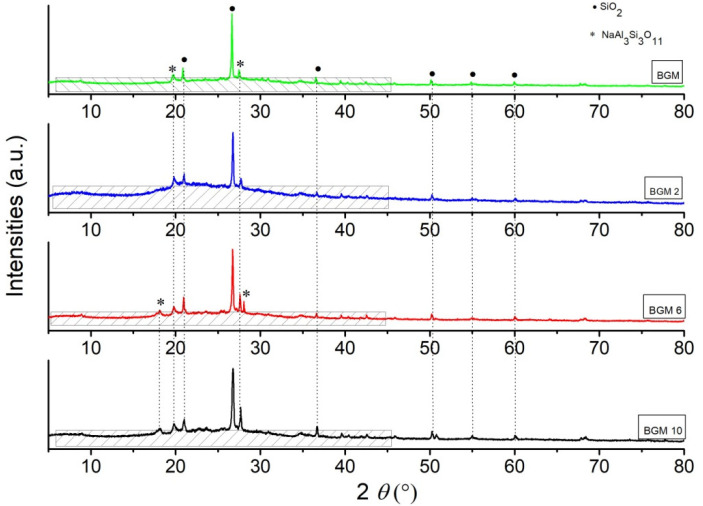
XRD diffraction patterns of BGM, BGM2, BGM6, and BGM10.

Phase analysis results obtained by XRD analysis of the synthesized hybrid GM samples are shown in Figure 2, and BGM samples are shown in Figure 3. Based on the X-ray diffraction results, it is clear that all samples consist of an amorphous part in the range from 10 to 40° 2*θ* and a semi-crystalline part, i.e., residual mineral phases that did not fully decompose in the geopolymerization process [22,23]. The identified peaks of crystalline phases belong to the SiO_2_ (quartz) phase and the Na_3_Si_3_O_11_ (paragonite) phase, and the residual brushite phase is not identified, which has most likely, fully reacted in an inorganic polymer matrix due the geopolymerization process.

Via the XRD method, the percentage of crystallinity was determined. The crystallinity of a polymer refers to the degree to which there are regions where the polymer chains are aligned, and some degree of stereoregularity is required for this to occur. The degree of crystallinity can be explained as the ratio of the sum of the deconvoluted crystalline parts over the sum of the crystalline and the amorphous deconvoluted parts [24]. The calculation of the degree of crystallinity is obtained by deconvolution in the Gaussian curves and it is performed with six curves for the crystalline part and shaded area curves of the amorphous part, which is presented in Figure 2 and Figure 3. The degree of the crystallinity of samples is calculated from the area of the crystalline peaks from the diffraction pattern Ac, and the area of the amorphous peaks of diffraction Aa, Equation (1); the results for the crystallinity calculations of the investigated samples are presented in Table 2 [25]:

Χc = Ac/(Aa + Ac)∙100%(1)

Based on the results presented in Table 2, all samples have between 49% and 75% approximately of the amorphous phase in a structure. Additionally, it is noticeable that the BGM2 and GM10 samples have above 70% of the amorphous phase in a structure, indicating that these two inorganic matrixes attach and incorporate organic polymers much better than other samples. The degree of crystallinity in samples varies between 25% and 50%, which shows that the proportion of crystalline phases refers to residual untransformed mineral phases originating from the raw clay that was used as a source of an aluminosilicate matrix [14,24]. This is in accordance with the material used, mostly untransformed grains of quartz and aluminosilicate minerals as found in the literature, and these materials can be said to be semi-crystalline polymer materials, where phosphate cement is most likely integrated into an amorphous matrix.

### 3.2. BET Analysis Results

**Figure 4:**
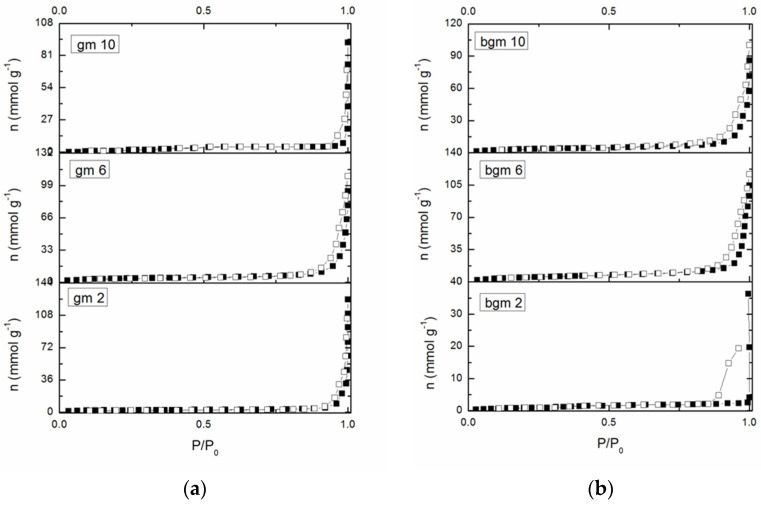
Nitrogen adsorption isotherm plot for (**a**) GM and (**b**) BGM samples. Solid symbols-adsorption; open symbols-desorption.

Nitrogen adsorption-desorption isotherms of GM2, GM6, and GM10 and BGM2, BGM6, and BGM10 are shown in Figure 4. According to the IUPAC classification, isotherms are type IV and with an H3 hysteresis loop, which is associated with mesoporous materials [25]. The specific surface, SBET, of all the samples lies between 6 and 19 m^2^g^−1^, and originates exclusively from the mesoporous structure of the samples (Smic~0, Vmic~0).

For the samples with a good adsorption capacity, this certainly originates from the functional groups of the organic compound and not from the surface of the geopolymer samples. The specific surface area is very small in samples GM 10 and BGM 2, which is in good correlation with the XRD results since these two samples have the highest content of the amorphous phase in their structure. Therefore, the SBET of these samples cannot be determined, nor can the other parameters be presented in the table. Since the surface area of these samples is very small, more precisely, in the case of 50% of the samples, we are talking about the geometric surface area, no pore distribution was performed. This indicates that the organic polymers attached to the surface of the geopolymer filled the pores, so these samples cannot be said to have open porosity.

### 3.3. DRIFT Analysis Results

**Figure 5:**
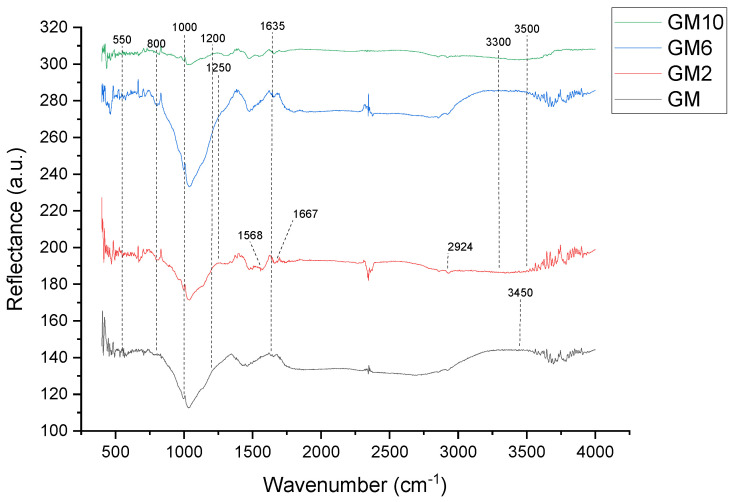
DRIFT spectra of GM, GM2, GM6, and GM10.

**Figure 6:**
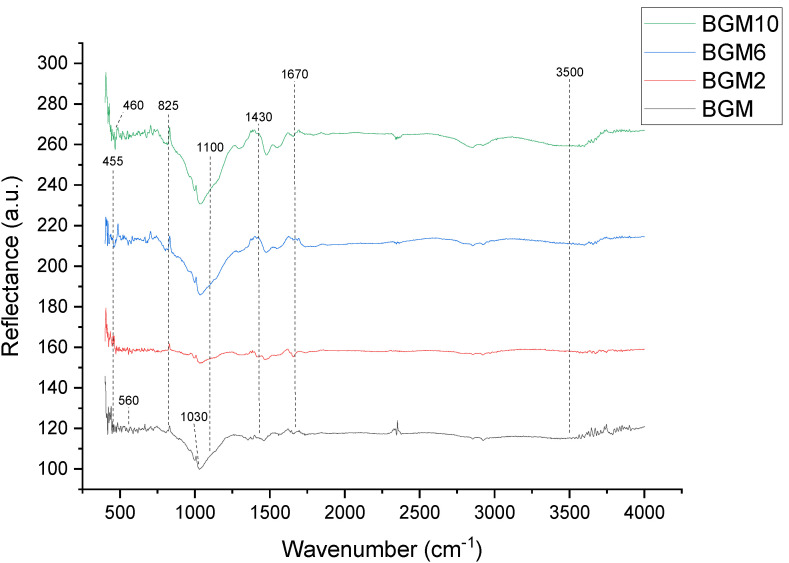
DRIFT spectra of BGM, BGM2, BGM6, and BGM10.

The DRIFT results, presented in Figure 5 and Figure 6, are used to determine and explain the functional groups of synthesized samples.

Figure 5 presents the results of the samples GM, GM2, GM6, and GM10. The characteristics for all the samples are the vibration bands with a maximum of ~3450 cm^−1^ and bands at 1635 cm^−1^, which are attributed to stretching vibrations and the deformation of physically adsorbed water molecules (H-O-H) on the surface. The presented spectra in Figure 5 of all samples show a strong peak at approximately 1000 cm^−1^, which is associated with the Si-O-Si asymmetric stretching vibration and fingerprints of polymerization [26]. In the part of the spectrum corresponding to wave numbers at 1000–1200 cm^−1^, there are two broad bands: the Si-O-Si stretching vibration and the Si-O and Si (Al)-O stretching vibration. The vibrational band with a maximum at approximately 1050 cm^−1^ is assigned to Si-O stretching of tetrahedra, in which Si is surrounded by three oxygen bridges and one non-bonding oxygen. The structure Si-O-X (X = Al, Si), the building blocks of geopolymers, and the shift of vibrational bands corresponding to the Si-O-X bond stretching toward the lower wavenumbers indicate the lengthening of the O-X bond of this structure and the reduction in the bond angle [26].

According to the literature, vibrations bands are attributed to secondary building units (SBUs) that are in the range of wave numbers from 800 to 550 cm^−1^. SBUs consist of fused SiO_4_ and AlO_4_ tetrahedral [27]. In the spectra of the samples, there is a maximum at the wave number ~710 cm^−1^, which can be attributed to the asymmetric stretching of Si-O-Al.

After impregnating the geopolymer with PEI, a group of new peaks appeared. In the sample GM2, the peaks at ~1568 and ~1667 cm^−1^ are related to the asymmetric bending vibration H-O-H and the bending vibration of N-H, respectively [28], while the peak ~2924 cm^−1^ is related to the stretching vibration of C-H [29]. The appearance of these peaks indicates that PEI is successfully loaded. A peak in the region of 3300–3500 cm^−1^ is attributed to amine N-H stretching. A weak peak at 1457 cm^−1^ is probably due to CH2 deformation. A peak in the region of 1020–1250 cm^−1^ can be attributed to C-N stretching of amines. The spectra show that the sample has both amines and silica features, thus implying that PEI was impregnated well.

In the samples with added brushite (Figure 6), the band appearing at about 560 cm^−1^ is characteristic of the brushite–metakaolin system and it can be attributed to the vibrations of the -P-O-Al-O- polymer molecules [8]. Additionally, the characteristic peaks are the two bands near 3500 cm^−1^ and 1670 cm^−1^, which are related to the presence of free or adsorbed water in all the samples. This indicates that the material contains sialon group and residual structural water, which is very common in clay [8].

The strong stretching vibration of Si-O-Si at 1100 cm^−1^ and 825 cm^−1^ can be found in all materials, which is assigned to asymmetrical stretching and symmetrical stretching, respectively [30]. With a bending vibration peak of Si-O-Si that appears at 455 cm^−1^, the band exhibiting at 460 cm^−1^ corresponds to the bending vibrations of the surface Si-OH groups [31]. The wide stretched band at about 1030 cm^−1^ can be related to the inclusions of PO4 tetrahedral units in the geopolymer system to form a -Si-O-Si-O-Al-O-Si-O-P-O- network. A characteristic peak for the Ca-O bond is observed at 1430 cm^−1^. The peaks that appeared at 1090 cm^−1^ corresponded to the antisymmetric stretching vibration of a phosphate group.

### 3.4. SEM-EDS Analysis

**Figure 7:**
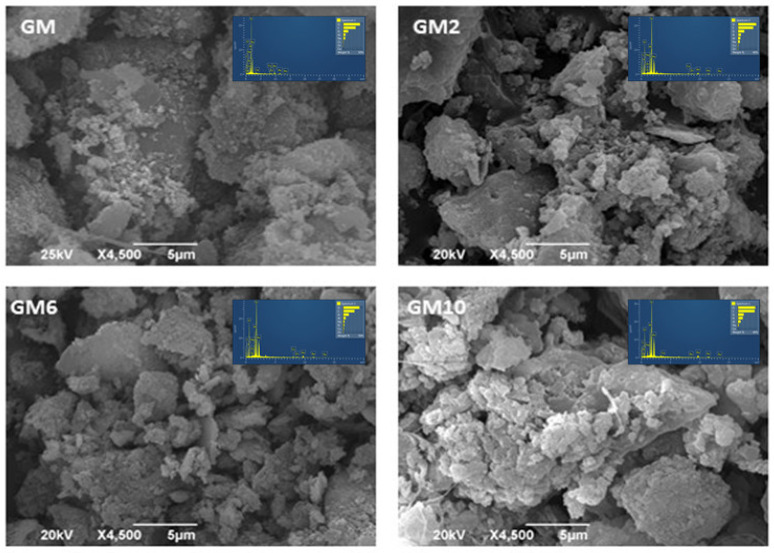
SEM-EDS microphotographs of GM, GM2, GM6, and GM10.

The scanning electron microscopy results show the morphological characteristics of the GM and BGM samples. Microphotographs of GM, GM2, GM6, and GM10 samples are shown in Figure 7, which presents the morphological features with EDS spectra of the synthesized hybrid polymer materials inserted.

All the sample figures indicate some slightly larger stacked grains of unreacted metakaolin that are between 5 µm and 20 µm in size. This is consistent with the activation process of aluminosilicates, such as metakaolin, where unreacted quartz and kaolin grains stay unreacted during the geopolymerization process [32]. The aluminosilicate matrix shows extremely small grains below the size of 1 µm, respectively, which are mutually agglomerated into larger spherical amorphous masses. Precisely these lighter amorphous fields represent the attached organic polymers whose agglomeration creates spherical masses up to 5 µm in size, where the agglomerates, as can be seen, clearly cover the aluminosilicate matrix. The highest coverage with the lightest covering on the SEM image was observed in sample GM10, where the highest amount of PEI was used, which is in correlation with the XRD data where the calculated amorphous phase amounts to about 72.83% (Table 2).

**Figure 8:**
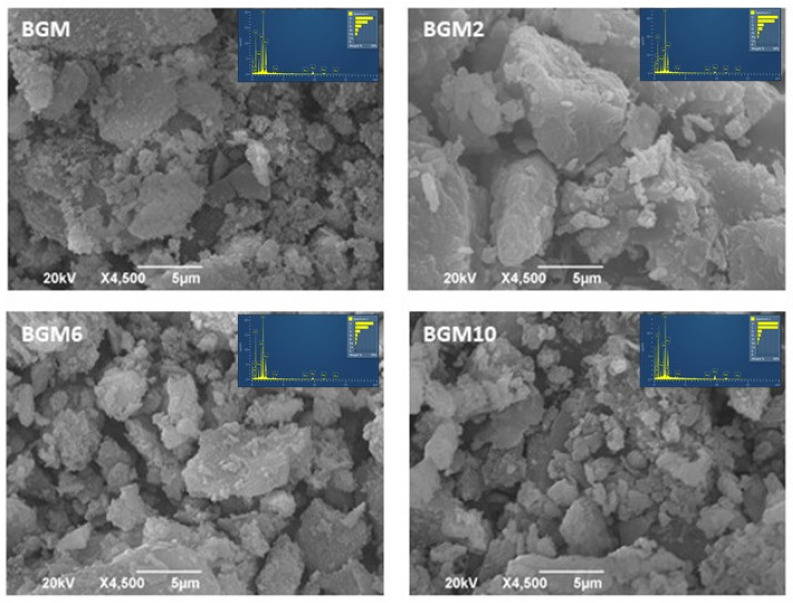
SEM microphotographs of BGM, BGM2, BGM6, and BGM10.

Figure 8 reveals quite a different morphology compared to the hybrid geopolymers without the addition of brushite in the structure. As it can be observed, the BGM sample consists of large, polymerized grains, which most likely represent brushite grains, which are partially reacted in the basic matrix with clay minerals, and represent unreacted grains in a semicrystalline matrix, which is in agreement with the xrd analysis. Additionally, on the surface of the grains in the basic matrix, amorphous geopolymer gel can be seen. Overlaying with an amorphous phase presents extremely small spherical forms that are slightly lighter than the basic matrix. As has been previously described, the BGM2 sample shows the brightest part where the polymer covers the entire semi-crystalline matrix, this is also confirmed by the XRD results, which has the highest calculated proportion of the amorphous phase in relation to all other samples and amounts to approximately 74.43% (Table 2). Microphotographs of the samples BGM6 and BGM10 with higher amounts of PEI in the structure show some larger unreacted semi-crystalline grains in the main matrix, which are approximately 10 µm in size, pretty non-uniform in their arrangement, and they overlap with the organic polymer. These results are also in correlation with the XRD data, where, for these two samples, the amount of the amorphous part is about 50% (Table 2). A BET analysis completely confirms the previously discussed results since the BET surface decreases with increasing amounts of the organic polymer and geopolymer.

EDS analysis spectra of obtained samples are inserted in the presented micrographs. Based on the EDS spectra analysis, it is noticeable that the Al:Si:Na ratio in the geopolymer materials is approximately 1:2:1. The highest amounts of N are in the samples BGM10 and GM10, which is in accordance with the synthesis procedure since 1,000,000 of PEI is attached in these samples. Additionally, the Ca:P ratio in the samples with brushite is approximately 1:1, which is in accordance with the literature data and also the synthesis of brushite [7,33].

### 3.5. TG Analysis

**Figure 9:**
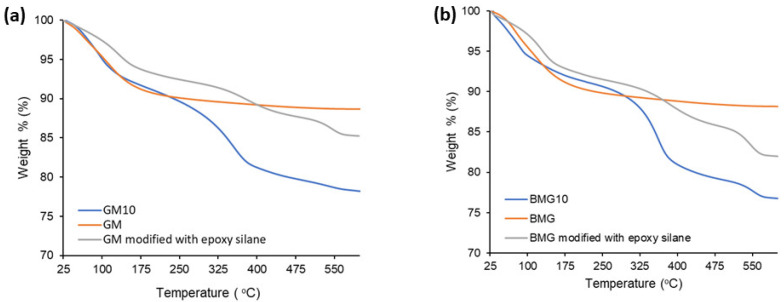
TG analysis of the (**a**) GM and (**b**) BGM samples.

The presence of epoxy silane and the PEI samples bound to the surface were determined by a TGA analysis. Figure 9 demonstrates the thermal behavior of the bare, epoxy-silane-modified and PEI (with a molecular weight of 1,000,000) covalently bonded GM and BGM composites. The total weight loss of GM, GM modified with epoxy silane, and GM10 was 11.1%, 14.7%, and 21.8%, respectively. The total weight loss of BGM, BGM modified with epoxy silane, and BGM10 was 11.7%, 17.7%, and 23.1%, respectively. As expected, the samples modified with epoxy silane showed a higher weight loss than the bare sample, while the samples to which PEI was covalently bonded showed a higher weight loss than the bare and epoxy-silane-modified samples. This indicates that epoxy silane and PEI successfully bonded to the surface of the composites.

### 3.6. CO_2_ Capture Analysis

The CO_2_ capacity results and N content of the samples are listed in Table 3. From the table, it can be seen that the amine-modified composites had a higher capture capacity than the bare samples. GM10 has the best CO_2_ capture capacity. GM10 has the best CO_2_ adsorption capacity among the prepared GM composites, while BGM10 has the best CO_2_ adsorption capacity among the BGM composites. As expected, the N content and CO_2_ adsorption capacity of the samples increased with the increasing PEI molecular weight.

**Figure 10:**
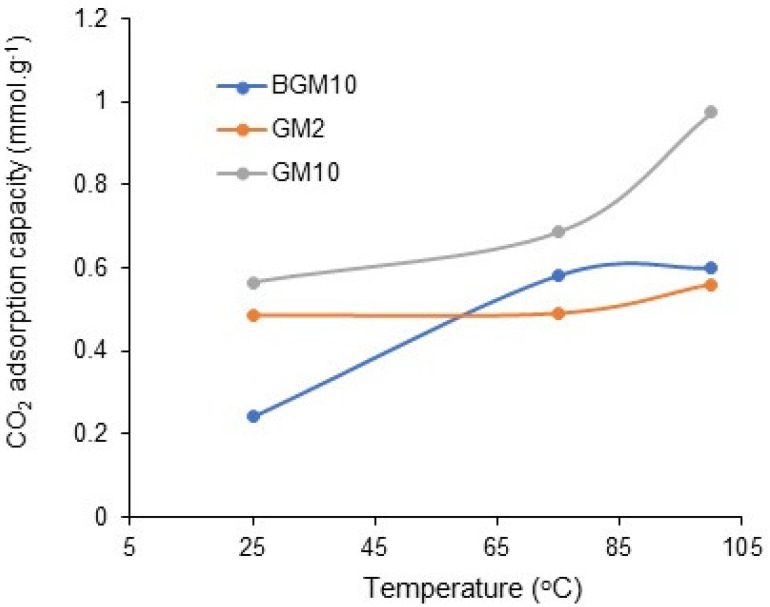
The CO_2_ adsorption capacities of BGM10, GM10, and GM2 composites at different temperature.

CO_2_ adsorption analyses of BGM10, GM10, and GM2 samples, which have the best adsorption capacity, were carried out at different temperatures (Figure 10). It was observed that the CO_2_ adsorption capacity of all three samples increased with the increase in temperature. This may be related to the endothermic reaction between amine molecules and CO_2_, or, with the increase in the temperature, the amines become more mobile and CO_2_ reaches more amines, thus increasing the CO_2_ capture capacity [34].

## 4. Conclusions

According to the XRD analysis, the degree of crystallinity of the samples and the area of the amorphous peaks of diffraction show that the samples GM10 and BGM2 had the highest % of amorphous phase (72.83 and 74.43, respectively), while the other samples had an approximately lower % of the amorphous phase, in region of ~48 to 59%.

Since the surface area of these samples is very small, this indicates that the organic polymers that are attached to the surface of the geopolymer filled the pores, so these samples cannot be said to have an open porosity.

The presented DRIFT spectra of all the samples show a strong peak at approximately 1000 cm^−1^, which is associated with the Si-O-Si or Si-O-Al stretching vibration, which is known as the “fingerprints” of polymerization. After impregnating the geopolymer with PEI, a group of new peaks appeared. The spectra show that the sample has both amines and silica features, implying that PEI was impregnated well.

The SEM analysis of the GM, GM2, GM6, and GM10 samples shows lighter amorphous fields where the agglomerates clearly cover the aluminosilicate matrix, and the highest coverage was observed in sample GM10 where the highest amount of PEI was used. This is in correlation with the XRD data where the calculated amorphous phase amounts to about 72.83% for the GM10 sample. Microphotographs of the samples BGM6 and BGM10 with higher amounts of PEI in their structures show some larger unreacted semi-crystalline grains in the main matrix, a non-uniform arrangement, and an overlap of the organic polymer.

Epoxy silane and PEI successfully bonded to the surface of the composites, due to the fact that the samples modified with epoxy silane showed a higher weight loss than the bare sample, while the samples to which PEI was covalently bonded showed a higher weight loss than both the bare and epoxy-silane-modified samples.

Considering the CO_2_ capture analysis, it was concluded that the amine-modified composites had a higher capture capacity than the bare samples. GM10 has the best CO_2_ capture capacity and the best CO_2_ adsorption capacity among the prepared GM composites, while BGM10 has the best CO_2_ adsorption capacity among the BGM composites. Given that inorganic geopolymer composites can be molded into various shapes, these newly designed materials offer the possibility of making good filter materials for the potential use as CO_2_ removers.

## Figures and Tables

**Table 1:** Prepared samples composition.

Sample	Metakaolin g	Brushite g	Weight Ratio of Composite to PEI	Weight Ratio of Composite to TMPTA
GM	100	/	/	/
GM2	100	/	/	1:10
GM6	100	/	1:1	/
GM10	100	/	1:1	/
BGM	98	2	/	/
BGM2	98	2	/	1:10
BGM6	98	2	1:1	/
BGM10	98	2	1:1	/

**Table 2:** Degree of crystallinity of investigated samples.

Sample	Degree of Crystallinity (%)	Amorphous (%)
GM	51.16 ± 5	48.84 ± 5
GM2	48.41 ± 5	51.59 ± 5
GM6	51.66 ± 5	48.34 ± 5
GM10	27.17 ± 5	72.83 ± 5
BGM	44.07 ± 5	55.93 ± 5
BGM2	25.56 ± 5	74.43 ± 5
BGM6	43.79 ± 5	56.21 ± 5
BGM10	40.86 ± 5	59.14 ± 5

**Table 3:** CO_2_ capture capacities at 75 °C and N contents of the composites.

Sample	CO_2_ Capacity (mmol·g^−1^)	N Content (mmol·g^−1^)
GM	0.217	2.34
GM2	0.481	3.94
GM6	0.283	5.74
GM10	0.685	5.99
BGM	0.247	4.56
BGM2	0.252	8.06
BGM6	0.295	3.50
BGM10	0.581	3.94

## Data Availability

Not applicable.
